# Investigation of Pathogenesis of H1N1 Influenza Virus and Swine *Streptococcus suis* Serotype 2 Co-Infection in Pigs by Microarray Analysis

**DOI:** 10.1371/journal.pone.0124086

**Published:** 2015-04-23

**Authors:** Xian Lin, Canhui Huang, Jian Shi, Ruifang Wang, Xin Sun, Xiaokun Liu, Lianzhong Zhao, Meilin Jin

**Affiliations:** 1 State Key Laboratory of Agricultural Microbiology, Huazhong Agricultural University, Wuhan, Hubei, P.R. China; 2 College of Veterinary Medicine, Huazhong Agricultural University, Wuhan, Hubei, P.R. China; College of Veterinary Medicine, CHINA

## Abstract

Swine influenza virus and *Streptococcus suis* are two important contributors to the porcine respiratory disease complex, and both have significant economic impacts. Clinically, influenza virus and *Streptococcus suis* co-infections in pigs are very common, which often contribute to severe pneumonia and can increase the mortality. However, the co-infection pathogenesis in pigs is unclear. In the present study, co-infection experiments were performed using swine H1N1 influenza virus and *Streptococcus suis* serotype 2 (SS2). The H1N1-SS2 co-infected pigs exhibited more severe clinical symptoms, serious pathological changes, and robust apoptosis of lungs at 6 days post-infection compared with separate H1N1 and SS2 infections. A comprehensive gene expression profiling using a microarray approach was performed to investigate the global host responses of swine lungs against the swine H1N1 infection, SS2 infection, co-infection, and phosphate-buffered saline control. Results showed 457, 411, and 844 differentially expressed genes in the H1N1, SS2, and H1N1-SS2 groups, respectively, compared with the control. Noticeably, genes associated with the immune, inflammatory, and apoptosis responses were highly overexpressed in the co-infected group. Pathway analysis indicated that the cytokine–cytokine receptor interactions, MAPK, toll-like receptor, complement and coagulation cascades, antigen processing and presentation, and apoptosis pathway were significantly regulated in the co-infected group. However, the genes related to these were less regulated in the separate H1N1 and SS2 infection groups. This observation suggested that a certain level of synergy was induced by H1N1 and SS2 co-infection with significantly stronger inflammatory and apoptosis responses, which may lead to more serious respiratory disease syndrome and pulmonary pathological lesion.

## Introduction

Swine influenza is a highly infectious acute respiratory viral disease of pigs that affects the respiratory tract and has considerable economic impacts [1]. Three main subtypes of swine influenza virus (H1N1, H3N2, and H1N2), with H1N1 as the predominant subtype, have circulated in pigs worldwide [2, 3]. In March 2009, a new swine-origin H1N1 influenza virus became a pandemic [4]. Pig infections with the new H1N1 virus have then been observed in multiple countries, showing that the pandemic H1N1 viruses have become established in swine populations [5–7]. Previous study has showed the new H1N1 viruses have spread from humans to pigs in China [8]. Swine influenza virus replication is mainly restricted to the epithelial cells in the respiratory tract, with the lung being the major target organ. Although it is a highly contagious virus for pigs and has high-morbidity but low-mortality rates, secondary complications would substantially worsen the illness and increase death rate [9]. In fact, swine influenza is one of the several significant contributors to the porcine respiratory disease complex (PRDC), which is caused by infection with more than one pathogen, such as the swine influenza virus and *Streptococcus suis* (*S*. *suis*) co-infection.

*S*. *suis* infections have been considered as a major problem worldwide in the swine industry and as a secondary agent of pneumonia, particularly in the past 20 years [10]. Among the 35 serotypes, serotype 2 (SS2) is generally considered as the most prevalent and virulent type [10]. *S*. *suis* infections in pigs often cause arthritis, meningitis, pneumonia, endocarditis, and septicemia with or without sudden death. Although *S*. *suis* is a major swine pathogen, it has been increasingly detected in wide range of mammalian species. Infections have been observed in humans in 2005 in China, which affected more than 200 people and had approximately 20% mortality [11]. In clinical cases, co-infections of swine influenza virus and *S*. *suis* in pigs often contribute to severe pneumonia and can increase the mortality. Co-infection outbreaks have been recently reported in England [12].

Recently, several studies on the pathogenesis of the co-infection of influenza virus and *Streptococcus pneumonia* have been performed using mouse models [13–15]. Pro- and anti-inflammatory (IL-6, IL-1β, TNF-α, and IL-10) molecules were remarkably elevated in the blood in influenza virus and *Streptococcus pneumonia* co-infected mouse [16]. However, fewer studies have examined swine influenza and *S*. *suis* co-infection in pigs, and its pathogenesis is not yet fully elucidated. In the present study, microarray assay was utilized to explore the global host responses of porcine lungs that suffered from H1N1 influenza virus, SS2, H1N1-SS2 co-infection, and phosphate-buffered saline (PBS) to enhance the understanding of the H1N1 and *S*. *suis* co-infection pathogenesis through a pig model. Stronger inflammatory and apoptosis responses were determined to be important contributors to the increased pathogenicity caused by swine H1N1 and SS2 co-infection. Our study would improve the understanding of the pathogenesis of H1N1 and SS2 co-infection in pigs.

## Materials and Methods

### Ethics statement

The research protocol of animal experiment in this study was approved by the Biological Studies Animal Care and Use Committee in Hubei Province, China.

### Viruses, bacteria, and cells

Viruses used in this study were A/swine/Hubei/101/2009 (H1N1) as previously described [17], and quantified as egg infectious dose 50 (EID_50_) in special pathogen-free chicken eggs. Madin-Darby canine kidney cells (MDCK) and Porcine kidney cells (PK-15) were obtained from the China Center for Type Culture Collection (CTCC, Wuhan, China) and cultured in Dulbecco's modified Eagle medium (DMEM) (Invitrogen, Carlsbad, CA) supplemented with 10% heat-inactived fetal bovine serum (FBS) (HyClone, Logan, UT), 100 units/mL penicillin and 100 μg/mL streptomycin (Invitrogen) at 37°C with 5% CO2. An SS2 strain, 05ZY, was isolated from the brain of a diseased piglet in Sichuan Province, China in 2005. Colony forming units (CFU) were counted through serial dilutions of bacteria on tryptic soy agar (TSA) plates supplemented with 5% (v/v) FBS.

### Infection experiment

The pigs used in the present study were raised in isolated facilities and determined to be negative for influenza virus antibody and SS2 antibody by analyzing the pig sera with hemagglutination inhibition test and enzyme-linked immunosorbent assay, respectively. The pigs were randomly divided into four groups with three pigs per group. The assigned groups were as follows: H1N1 infection alone (H1N1), SS2 infection alone (SS2), H1N1 and SS2 co-infection (H1N1-SS2) and PBS control. Considering the animal welfare and avoiding the waste, H1N1 and control groups were the same as those used in our previous study [17], because the two experiments were performed at the same time and under same conditions. Each group was housed in an isolated room. At 35 day of age, the H1N1 and H1N1-SS2 groups were inoculated intranasally with 2 mL of 10^7.0^ EID_50_/mL of the H1N1 swine influenza virus, and the other pigs were inoculated with PBS. On day 3 of the experiment (3 d after H1N1 challenge), the SS2 and H1N1-SS2 groups were inoculated intranasally with 2 mL of PBS containing 10^6.0^ CFU/mL SS2 strain ZY05. The H1N1 and control groups were treated similarly with PBS. Considering the biological features of swine H1N1 virus and to be much closer to the natural infections, we performed the H1N1 infection first and then SS2 infection. All pigs were monitored daily for rectal temperature and clinical signs. The nasal swabs were obtained from the right nostrils every other day using sterile cotton swabs. On day 6 of the experiment, all pigs were humanely euthanized by intravenous injection of 60 mg/kg body weight sodium pentobarbital. Lungs from all groups were immediately obtained. The macroscopic lesions of lung tissues were estimated visually, and scored blindly by a pathologist. Lesion areas in left caudal lobes of the lungs were immediately frozen and stored in liquid nitrogen after collection until RNA extraction, and another area in the same lobes was used for isolating the virus and bacteria. Lesion areas in right caudal lobes were fixed in formalin and embedded in paraffin, sectioned at 5μm, and stained with hematoxylin and eosin for further histopathologic evaluation. Slides were randomized, and read blindly by an experienced pathologist. At least 6 sections per lung from each group were examined microscopically for tissue damage, necrosis, apoptosis, and inflammatory cellular infiltration.

### Virus and bacteria isolation

At specified time points, the collected nasal swabs were inserted into vials containing 1.5 mL of sterile PBS supplemented with 100 U/mL penicillin and streptomycin. After thorough vortex, the vials were centrifuged at 12,000 rpm at 4°C for 10 min. The supernatants were collected, and the virus titer was quantified as EID_50_. To prevent cross-contamination, separate sterile instruments were used to collect the lungs of pigs from each group, which were homogenized in sterile PBS and then centrifuged to collect the supernatant. Virus titer was quantified as EID_50_, and SS2 content was determined by incubation on TSA plates supplemented with 5% (v/v) FBS for CFU counting.

### Agilent microarray experiment

The same areas in the lungs from each group were sampled for RNA extraction using TRIzol (Invitrogen, Carlsbad, CA) and RNaeasy MiNi Kit (QIAGEN) according to the manufacturer’s instruction. The microarray experiments were performed as previously described [17]. RNA labeling and hybridization were performed using a commercial Agilent array (Shanghaibio, China) that followed the standard one-cycle protocol according to the manufacturer’s instructions. Transcriptional profiles were assessed using Agilent 4x44K Porcine Oligo Microarray. The hybridization and scanning of the arrays were conducted using a G2565BA Scanner (Agilent Technologies, Palo Alto, CA) according to the standard protocol. The hybridization data were normalized using Agilent Feature Extraction Software. ANOVA was used to analyze the data. The raw and processed microarray data of H1N1 and control groups used here were described as our previous study [17], because these two experiments were performed under same conditions. All differentially expressed (DE) genes were then examined using hierarchical cluster (Ver. 3.0) and TreeView (Ver. 1.1.1) analyses [18]. Genes with significant similarities (greater than 70%) to transcripts in the nr database which were functionally annotated based on BLASTX searches were selected for GO and pathway analyses using MAS 3.0 software, which is based on DAVID and KEGG databases (CapitalBio, Beijing, China) [19]. All DE genes were identified by setting a fold change (FC) > 1.5 and p < 0.05 as the criteria.

### Real-time quantitative PCR (RT-qPCR)

RT-qPCR was used to validate the expression data. RNA was extracted using TRIzol (Invitrogen). RNA (0.5 μg) was reversely transcribed in a 20μl reaction mixture containing 2 μl avian myeloblastosis virus (AMV) buffer, 50 pM Oligo18T, 0.5 mM dNTPs, 10 U RNase inhibitor and 10 U AMV reverse transcriptase (TAKARA, Japan), and the gene expression was monitored through a Power SYBR Green PCR master mix kit (Applied Biosystems) with corresponding primers. The fluorescence signals were measured using the ABI ViiA7 PCR system (Applied Biosystems). GAPDH was used as the internal control, and all gene expressions were normalized to GAPDH. The primers used are listed in S1 Data.

### Microarray Data Accession Number

The raw and processed data in this study have been deposited in NCBI’s Gene expression omnibus and are accessible through GEO Series accession number GSE60172.

### Statistical analysis

Standard two-sample t-tests were used to compare virus titers, gene expressions, lung pathological damage score, and the apoptosis rate among infection groups. All data are expressed as the mean ± SEM. A value of P < 0.05 was considered statistically significant.

## Results

### Clinical signs and pathological evaluation

**Fig 1:**
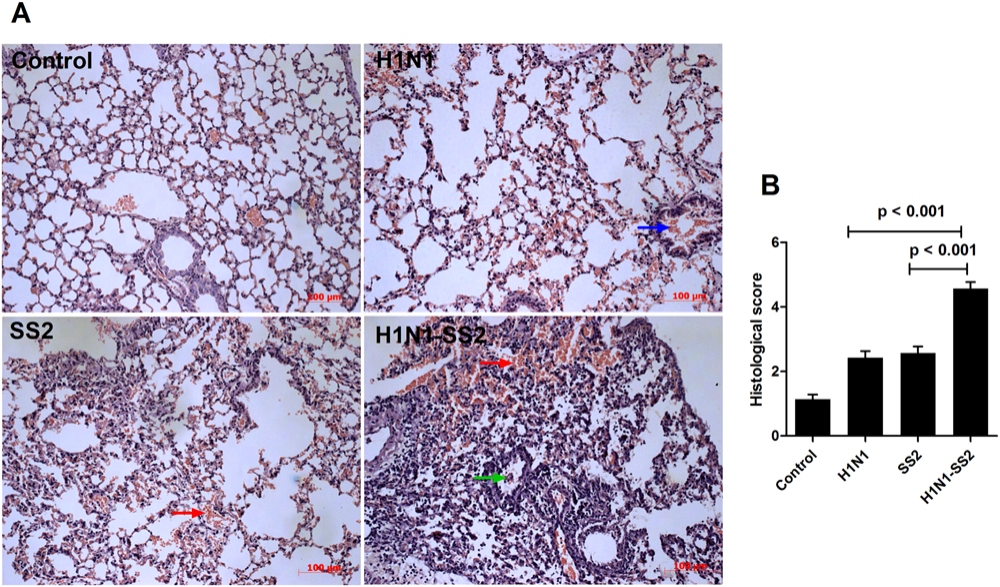
Histopathologic changes in lungs of infected pigs. Pigs were PBS-control infected or infected with H1N1, SS2 and H1N1-SS2 as described in methods. On day 6 of the experiment, lungs were removed and underwent Hematoxylin and eosin stain. (A) The microscopic lesions of lung tissues from each group showed different extent of acute pneumonia with pathological changes: alveolar wall thickening, bleeding (red arrow), debris in the lumen (green arrow), erythrocyte effusion (blue arrow), and the accumulation of inflammatory cells. (B) Histological score of sections of lungs in pigs from each group were showed as mean ± SEM by two-tailed Student’s t-test. *P*-value less than 0.05 was considered to represent a statistically significant difference.

To assess the infection process, clinical signs were observed daily starting from 1 d before challenge (day 0) (Table 1). The H1N1 and H1N1-SS2 groups experienced fever, which started on day 1, reaching to 40.8°C and 40.9°C, respectively, on day 3. In the H1N1 group, coughing, nasal discharge, and anorexia were observed. Clinical signs recovered to normal on day 6. However, in the H1N1-SS2 co-infection group, in addition to the signs observed in H1N1 group, pigs also exhibited high fever (41.5°C, 41.8°C, and 41.5°C) on day 6. Moreover, limp and arthrocele, which are the typical symptoms of SS2 infection, were observed. In the SS2 infection group, all pigs did not show any abnormal sign before inoculating with SS2. However, 3 d after SS2 infection (day 6), the SS2 group exhibited typical SS2 infection symptoms, such as lameness, joint swelling, lethargy to different extents, and one pig exhibited fever over 40.5°C. Notably, the H1N1-SS2 co-infection group exhibited more severe symptoms compared with the SS2 infection alone group. To investigate pathological damage to lungs of infected pigs, pigs were euthanized on day 6, and the macroscopic lesions of lungs tissues were estimated visually (S2 Data). Much more serious lung pathological damage was caused by co-infection, including hyperemia, necrosis, bleeding, and consolidation, compared with separate infections. Microscopic pathological changes in the lungs were also evaluated (Fig 1A, S3 Data (Control), S4 Data (H1N1), S5 Data (SS2), and S6 Data (H1N1-SS2)). All groups except the control exhibited different degrees of pathological damage. Extensive lung damages (that were, extensive cellular infiltrates, bleeding, and cellular debris) were observed in H1N1-SS2 group. However, H1N1 and SS2 groups showed lesser lung damages. These observations were supported by blinded histological scoring (Fig 1B). These results demonstrated that H1N1 and SS2 co-infection increased the pathogenicity.

**Table 1:** Clinical signs after infection in each group.

Group	Clinical signs	
	Days 1–3	Days 4–6
Control	N.	N.
H1N1	Fever (3/3), cough (1/3), sneeze (1/3), snotty (2/3).	Fever (0/3), cough (1/3), sneeze (0/3), snotty (0/3).
SS2	N.	Fever (2/3), depressed (2/3), limp (2/3), arthrocele (1/3).
H1N1-SS2	Fever (3/3), cough (1/3), sneeze (1/3), snotty (1/3).	High fever (2/3), hypothermia (1/3), limp (2/3), recumbent (1/3), arthrocele (2/3), cough (1/3), sneeze (2/3), snotty (0/3).

Clinical signs in each infection group were monitored daily. N: No obvious clinical sign was observed. X/X refers to morbidity/total.

### Virus and bacteria isolation

**Fig 2:**
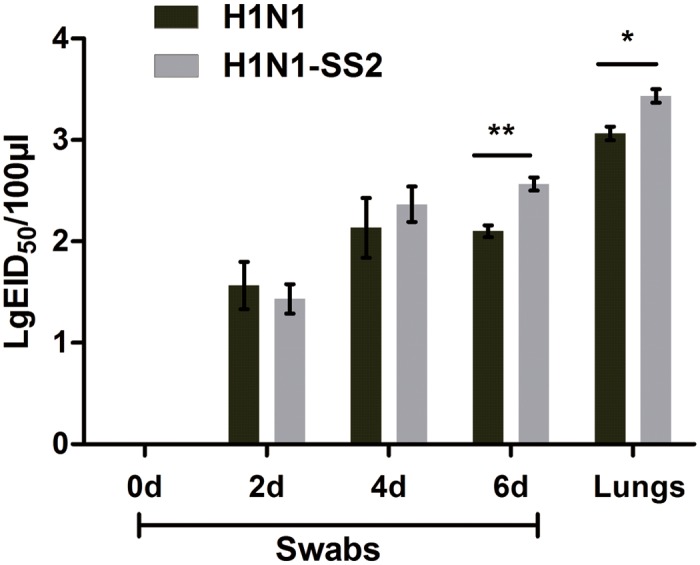
Virus isolation of nasal swabs and lungs in each group. Swabs were collected from the right nostrils of pigs from each group every other day. The obtained nasal swabs were inserted into vials containing 1.5 mL of sterile PBS. Supernatants were collected and viruses were qualified as LgEID_50_. At the day 6 of the experiment, all pigs were humanely euthanized and lungs were collected and homogenized in sterile PBS and then centrifuged to collect the supernatant. Virus titer was quantified as LgEID_50_. Data were showed as mean ± SEM by Student’s t-test. *P*-value less than 0.05 was noted with a single-asterisk and p-value less than 0.01 was noted with a double-asterik.

The swabs, which were used for virus isolation, were collected from all pigs every 2 d after inoculation. As expected, virus were isolated from the pigs in the H1N1 and H1N1-SS2 groups on days 2, 4, and 6 (Fig 2). The virus titer in the H1N1-SS2 groups was slightly but significantly higher than that in the H1N1 group on day 6. On day 6 after inoculation, all pigs were humanely euthanized, and viruses in lungs were also detected in both the H1N1 and H1N1-SS2 groups. The virus titer in the H1N1-SS2 group was also significantly higher than that in H1N1 group (Fig 2). No virus was isolated from the SS2 and control groups. However, no significant difference was observed on the bacterial loads in the lungs between the H1N1-SS2 and SS2 groups (data are not shown). No bacteria were detected in the H1N1 and control groups.

### Global gene expression modified by each infection

**Fig 3:**
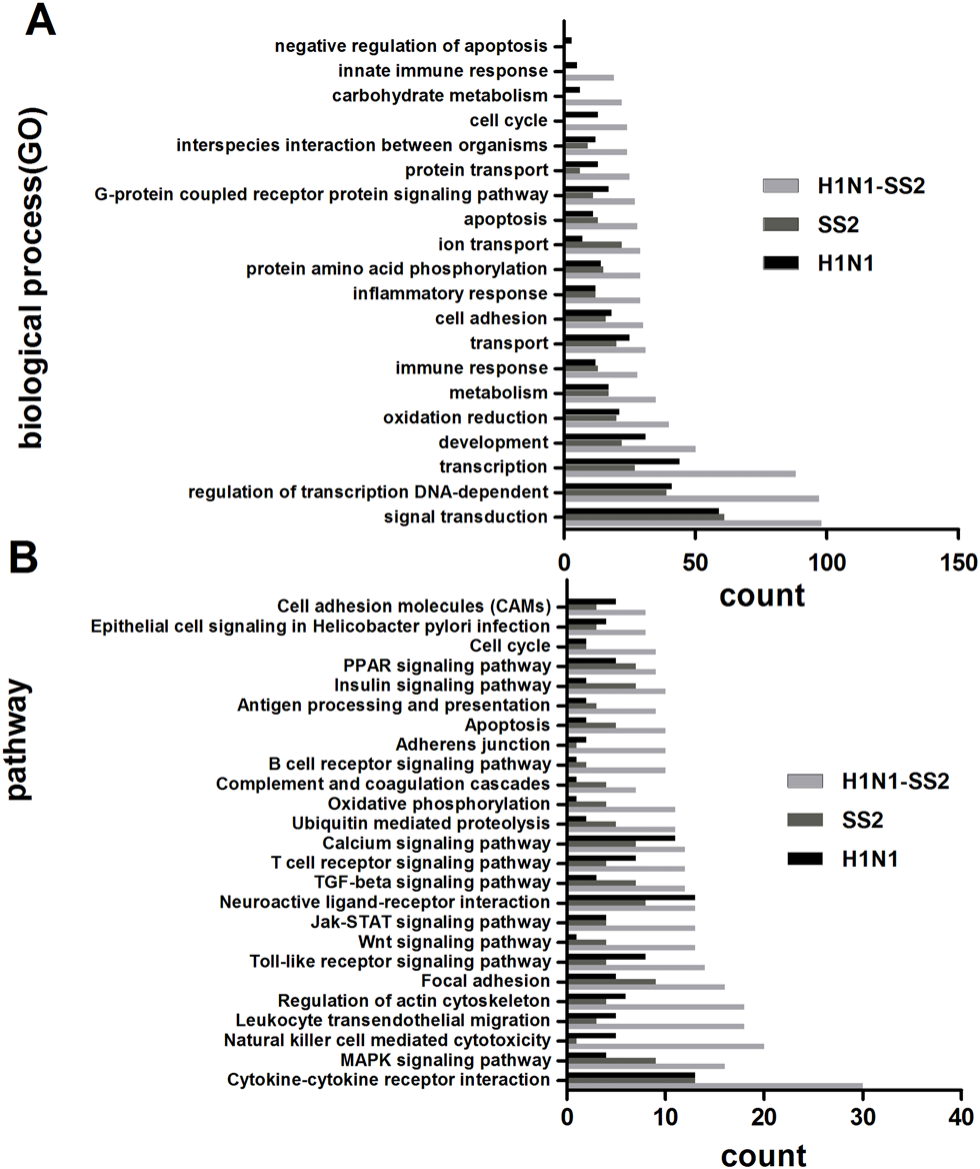
Characterization of the differential expression of genes. (A) Categories of genes based on biological process GO term in each group. Only the top20 terms based on the gene numbers are showed. (B) Clustered pathways of the DE genes by KEGG analysis. Only the pathways containing more genes are presented. Many categories shared the same genes.

To further understand the H1N1 influenza virus and SS2 co-infection pathogenesis in pigs, Agilent microarray assay was performed to analyze the gene expression in the lungs from each group. The expression profiles of porcine lungs of the infected groups were compared with that in the control group. After quantile normalization and statistical analyses, 1314, 1353, and 2652 transcripts were identified to be different expression in H1N1, SS2, and H1N1-SS2 groups, respectively. These transcripts represented the corresponding 457 genes (230 upregulated and 227 downregulated), 411 genes (199 upregulated and 212 downregulated), and 844 genes (597 upregulated and 247 downregulated), respectively. MAS3.0 was used to analyze the functions of the DE genes. Majority of the DE genes were associated with signal transduction, transcription, development, immune response, cell adhesion, inflammatory response, apoptosis, innate immune response, and oxidation reduction (Fig 3A). Interestingly, inflammatory response and apoptosis were more intensely activated in the H1N1-SS2 group compared with the H1N1 and SS2 groups, indicating that they may have important roles in the infection process. To gain insight into the different biological processes of each infection, pathway analysis was also performed on the DE genes. Those containing more genes were selected for the analysis (Fig 3B), which showed that more DE genes were clustered in the immune and inflammation response (cytokine–cytokine receptor interaction, MAPK signaling, Toll-like receptor (TLR) signaling, JAK-STAT signaling pathway, antigen processing and presentation, and Complement and coagulation cascades) and apoptosis in the H1N1-SS2 group than the two other infection groups.

### Genes related to inflammatory response

**Fig 4:**
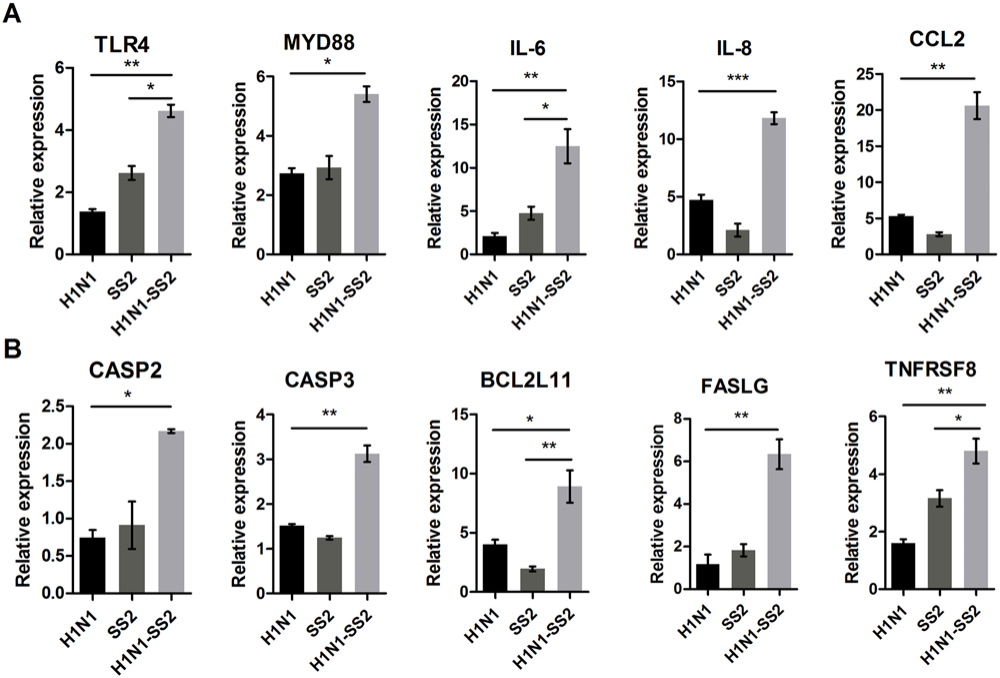
Expression of genes related to inflammation and apoptosis by qRT-PCR. RNA was extracted from lungs of each infection group, and was reversely transcribed. mRNA levels were measured by real-time PCR. (A) Changes of genes related to proinflammatory response. (B) Changes of genes related to apoptosis. Data were showed as mean ± SEM and were representatives of two independent experiments. Significant levels were analyzed by T-test.

The DE genes associated with inflammatory responses were examined to further investigate the different gene expression. H1N1-SS2 co-infection increased the expression of more cytokine–cytokine receptor interaction genes compared with the other two infection groups (Fig 3B and Tables 2, 3 and 4). These genes included interferon receptors IFNAR1 (1.74-fold), IFNAR2 (2.27-fold), and IFNGR1 (1.73-fold). Several interleukin receptors were also significantly upregulated, including IL13RA1 (1.65-fold), IL1R2 (4.56-fold), and IL4R (2.15-fold). In addition, co-infection significantly regulated the mRNA expression of several chemokine and chemokine receptor genes, including CCL3 (1.62-fold), CX3CL1 (1.7-fold), CXCL16 (0.56-fold), CCR1 (2.23-fold), and CXCR4 (1.55-fold). Although the pro-inflammatory cytokine TNF-α was not significantly regulated, five tumor necrosis factor receptor superfamily genes were significantly regulated in the co-infection group (Table 4). To describe the inflammatory responses, several genes related to inflammation were tested by RT-PCR (Fig 4A). It indicated that TLR4 and MYD88 were significantly increased in H1N1-SS2 infection group, which play important roles in TLR signaling. IL-8, CCL2, and IL-6 were also remarkably increased in the co-infection group.

**Table 2:** The DE genes associated with immune and inflammatory responses in H1N1 group.

Functional classification	Description	Gene symbol	Gene ID	Fold change
**Immune response**	complement component 6	C6	100037952	2.66
	toll-like receptor 3	TLR3	100037937	-2.54
	ring finger protein 125, E3 ubiquitin protein ligase	RNF125	100517591	2.19
	CD59 molecule, complement regulatory protein	CD59	397347	2.00
	CD1d molecule	CD1D	100124526	-1.93
	chemokine (C-C motif) receptor-like 1	CCRL1	100037292	1.86
	peroxidasin homolog (Drosophila)	PXDN	100516076	1.72
	*Rep*:*CD274 molecule CD274*, *mRNA- Homo sapiens*	CD247	397302	1.71
	lymphocyte cytosolic protein 2 (SH2 domain containing leukocyte protein of 76kDa)	LCP2	100511843	1.70
	toll-like receptor 1	TLR1	396607	1.65
	GTP binding protein overexpressed in skeletal muscle	GEM	404772	-1.63
	CD4 molecule	CD4	404704	1.58
	toll-like receptor 6	TLR6	396621	1.58
**Inflammatory response**	sphingosine-1-phosphate receptor 3	S1PR3	100154607	2.29
	*Rep*: *transforming growth factor*, *beta 2- Macaca mulatta*	TGFB2	397084	-2.18
	interleukin 17D	IL17D	100738902	2.16
	chemokine (C-C motif) ligand 2	CCL2	397422	-1.99
	interferon (alpha, beta and omega) receptor 1	IFNAR1	396658	1.89
	vascular endothelial growth factor C	VEGFC	100127470	-1.87
	CD163 molecule	CD163	397031	-1.84
	interleukin 2	IL2	396868	1.71
	toll-like receptor adaptor molecule 2	TICAM2	100520421	1.66
	mitogen-activated protein kinase 12	MAPK12	574062	-1.65
	chemokine (C-C motif) ligand 20	CCL20	553951	1.61
	chemokine (C-X-C motif) ligand 2	CXCL2	414904	1.59
	tumor necrosis factor receptor superfamily, member 1B	TNFRSF1B	100037306	1.58
	inhibitor of kappa light polypeptide gene enhancer in B-cells, kinase gamma	IKBKG	100127355	-1.52
	toll-interleukin 1 receptor (TIR) domain containing adaptor protein	TIRAP	100514174	-1.71

The DE genes associated with immune and inflammatory responses were assigned based on GO term and manual annotation. Manual annotations were listed in italics. Many genes with multiple functions were only listed in one category. Gene ID refers to NCBI gene ID in the study.

**Table 3:** The DE genes associated with immune and inflammatory responses in SS2 group.

Functional classification	Description	Gene symbol	Gene ID	Fold change
**Immune response**	protein C receptor, endothelial	PROCR	654289	2.86
	chitinase 1 (chitotriosidase)	CHIT1	100512552	2.50
	C-type lectin domain family 5, member A	CLEC5A	397050	-2.21
	chitinase, acidic	CHIA	100156433	1.99
	Fc fragment of IgA	FCAR	100144539	1.99
	cytotoxic T-lymphocyte-associated protein 4	CTLA4	397286	1.80
	dipeptzzidyl-peptidase 8	DPP8	100155833	-1.53
	interleukin 1 receptor, type II	IL1R2	100628112	14.28
	CD14 molecule	CD14	100037938	5.23
	CD163 molecule	CD163	397031	3.60
	A1 adenosine receptor	ADORA1	606743	3.08
	histone deacetylase 9-like	HDAC9	100523163	-3.07
	interleukin 2	IL2	396868	-2.50
	sialoadhesin	SIGLEC1	397623	-2.22
	kinase insert domain receptor (a type III receptor tyrosine kinase)	KDR	397311	-2.22
	insulin-like growth factor binding protein 4	IGFBP4	100144490	2.14
**Inflammatory response**	v-kit Hardy-Zuckerman 4 feline sarcoma viral oncogene homolog	KIT	396810	2.10
	interleukin 17D	IL17D	100738902	2.07
	erythropoietin receptor	EPOR	397554	-2.07
	vascular endothelial growth factor A	VEGFA	397157	-1.91
	erythropoietin	EPO	397249	1.85
	chemokine (C-X-C motif) ligand 12	CXCL12	494460	-1.77
	VCP-interacting membrane protein	SELS	100151836	1.67
	neutrophil cytosolic factor 4, 40kDa	NCF4	100152055	1.64
	KIT ligand	KITLG	397509	-1.60
	*Rep*: *transforming growth factor*, *beta 2- Macaca mulatta*	TGFB2	397084	-1.60
	toll-like receptor 9	TLR9	397007	1.58
	vacuolar protein sorting 45 homolog (S. cerevisiae)	VPS45	100514766	1.56

The DE genes associated with immune and inflammatory responses were assigned based on GO term and manual annotation. Manual annotations were listed in italics. Many genes with multiple functions were only listed in one category.

**Table 4:** The DE genes associated with immune and inflammatory responses in H1N1-SS2 group.

Functional classification	Description	Gene symbol	Gene ID	Fold change
**Immune response**	CD1d molecule	CD1D	100124526	3.99
	A1 adenosine receptor	ADORA1	606743	3.37
	chitinase 1 (chitotriosidase)	CHIT1	100512552	3.02
	*PREDICTED*: *Sus scrofa guanylate binding protein 4 (GBP4)*	GBP4	100155195	2.91
	nuclear factor, interleukin 3 regulated	NFIL3	100153822	2.53
	SAM domain and HD domain 1	SAMHD1	100625064	2.41
	CD72 molecule	CD72	100038011	2.39
	vav 1 guanine nucleotide exchange factor	VAV1	100519821	2.41
	bone morphogenetic protein 2	BMP2	100157103	-1.88
	mitochondrial antiviral signaling protein	MAVS	100037290	1.74
	bone morphogenetic protein receptor, type IB	BMPR1B	396691	-1.72
	Fc fragment of IgG, low affinity IIIb, receptor (CD16b)	FCGR3B	397684	1.71
	DEAD (Asp-Glu-Ala-Asp) box polypeptide 46	DDX46	100626833	-1.69
	hepcidin antimicrobial peptide	HAMP	397207	1.58
	granzyme A (granzyme 1, cytotoxic T-lymphocyte-associated serine esterase 3)	GZMA	100526762	-1.57
	B-cell linker	BLNK	100152350	1.56
	ferritin, heavy polypeptide 1	FTH1	397030	1.50
**Inflammatory response**	CD163 molecule	CD163	397031	4.98
	CD14 molecule	CD14	100037938	4.96
	*Rep*: *IL-1 receptor 2-Bos taurus Bovine*, *partial 98%*	IL1R2	100628112	4.56
	neutrophil cytosolic factor 4, 40kDa	NCF4	100152055	3.34
	myeloid differentiation primary response gene (88)	MYD88	396646	2.32
	oncostatin M receptor	OSMR	100519398	2.31
	toll-like receptor 9	TLR9	397007	2.27
	chemokine (C-C motif) receptor 1	CCR1	414374	2.24
	interferon (alpha, beta and omega) receptor 2	IFNAR2	100533555	2.23
	interleukin 4 receptor	IL4R	397614	2.15
	neutrophil cytosolic factor 1	NCF1	100134857	2.10
	toll-like receptor adaptor molecule 2	TICAM2	100520421	2.00
	kininogen 1	KNG1	396568	-2.08
	erythropoietin	EPO	397249	1.94
	bone morphogenetic protein 2	BMP2	100157103	-1.88
	fms-related tyrosine kinase 3 ligand	FLT3LG	100322867	-1.85
	lipopolysaccharide binding protein	LBP	397303	1.81
	vascular endothelial growth factor A	VEGFA	397157	-1.81
	allograft inflammatory factor 1	AIF1	397271	1.80
	toll-like receptor 4	TLR4	399541	1.79
	scavenger receptor for phosphatidylserine and oxidized low density lipoprotein	CXCL16	396735	-1.78
	SMAD family member 1	SMAD1	397016	1.75
	interferon (alpha, beta and omega) receptor 1	IFNAR1	396658	1.74
	nuclear factor of kappa light polypeptide gene enhancer in B-cells inhibitor, alpha	NFKBIA	406188	1.74
	interferon gamma receptor 1	IFNGR1	100152238	1.73
	bone morphogenetic protein receptor, type IB	BMPR1B	396691	-1.72
	chemokine (C-X3-C motif) ligand 1	CX3CL1	100621027	1.70
	activin A receptor, type IIA	ACVR2A	397282	1.68
	neutrophil cytosolic factor 2	NCF2	100142665	1.68
	toll-interleukin 1 receptor (TIR) domain containing adaptor protein	TIRAP	100514174	-1.66
	interleukin 13 receptor, alpha 1	IL13RA1	397615	1.65
	*chemokine (C-C motif) ligand 3-like 1*	CCL3	494459	1.62
	anti-Mullerian hormone receptor, type II	AMHR2	100154297	-1.58
	B-cell linker	BLNK	100152350	1.56
	bone morphogenetic protein receptor, type IB	BMPR1B	396691	1.56
	chemokine (C-X-C motif) receptor 4	CXCR4	396659	1.55
	interleukin 17D	IL17D	100738902	1.51
	interleukin 6 signal transducer (gp130, oncostatin M receptor)	IL6ST	100037294	1.50
	NADPH oxidase heavy chain subunit	CYBB	397108	1.50
	signal transducer and activator of transcription 5B	STAT5B	397340	1.50

The DE genes associated with immune and inflammatory responses were assigned based on GO term and manual annotation. Manual annotations were listed in italics. Many genes with multiple functions were only listed in one category.

### Robust apoptosis response to H1N1-SS2 co-infection

**Fig 5:**
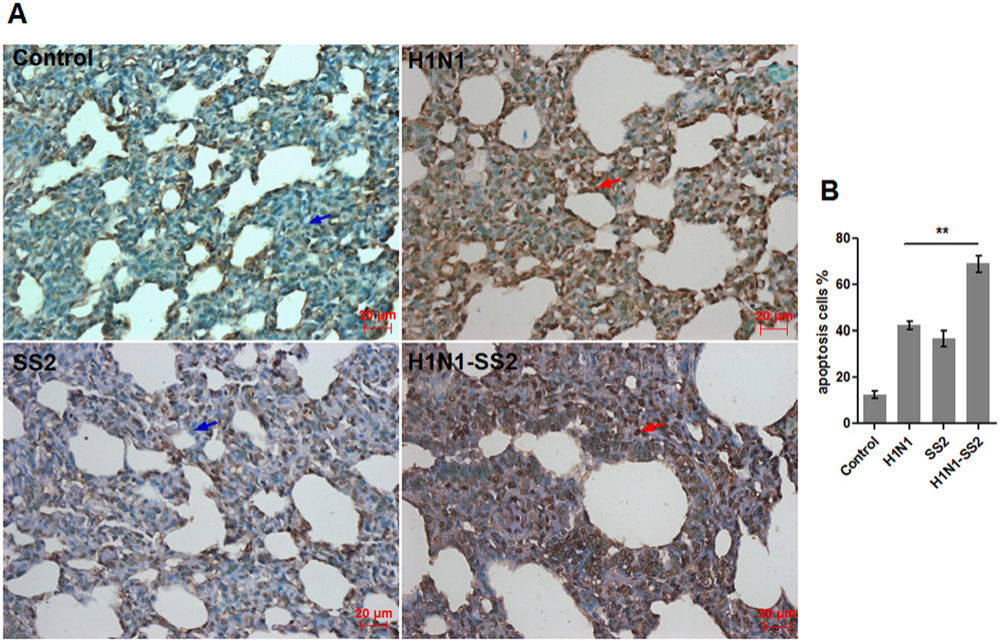
Apoptosis induced by H1N1 virus, SS2, H1N1-SS2 and PBS control. Histological sections of lungs from each group were evaluated for apoptosis by TUNEL test. (A) The infected groups showed varying degrees of apoptosis. Brown ovals (red arrows) indicate apoptotic nucleus. Blue ovals (blue arrows) indicate normal nucleus. (B) Statistical analysis of apoptosis rate in each infection group. Data was showed as mean ± SEM. T-test was used to analyze the significance.

The microarray analysis results showed that a number of genes involved in apoptosis were markedly upregulated to various extents in the H1N1-SS2 group (Table 5). These genes included the tumor necrosis factor receptor (TNFR) superfamily members (TNFRSF10A, TNFRSF1B, and TNFRSF8), genes involved in the activation cascade of caspase that is responsible for apoptosis (CASP2, CASP3, and CASP4) and BCL2-like genes (BCL2L11 and BCL2L14). qRT-PCR also demonstrated co-infection significantly increased expressions of CASP2, CASP3, BC2L11, FASLG, and TNFRSF8 (Fig 4B). The markedly expressed apoptosis-associated genes in H1N1 and SS2 groups were relatively lesser in the H1N1-SS2 group. Thus, apoptosis in each group was further investigated. Terminal deoxynucleotidyl transferase dUTP nick end labeling (TUNEL) was used to detect cell apoptosis in the lungs from each group. Fig 5A showed that the H1N1 and SS2 infections caused significant apoptosis, compared with the control. However, H1N1-SS2 co-infection notably triggered the significantly more cell apoptosis rate compared with the two other infections (Fig 5B). This result was consistent with the modification of the significantly regulated genes involved in apoptosis by each infection (Table 5). The result suggested that abundant cell apoptosis induced by H1N1 and SS2 co-infection would contribute to the enhanced virulence.

**Table 5:** The DE genes associated with apoptosis in each infection group.

Group	Description	Gene symbol	Gene ID	Fold change
H1N1	C1D nuclear receptor corepressor	C1D	100515656	4.23
	cytoplasmic FMR1 interacting protein 2	CYFIP2	100523290	1.93
	*Rep*: *baculoviral IAP repeat-containing protein 2*	BIRC2	100622859	-1.85
	RING1 and YY1 binding protein	RYBP	100526249	-1.71
	lymphotoxin beta (TNF superfamily, member 3)	LTB	100155581	1.63
	lipopolysaccharide-induced TNF factor	LITAF	100518302	-1.62
	tumor necrosis factor receptor superfamily, member 1B	TNFRSF1B	100037306	1.58
	sulfatase 1	SULF1	100152427	-1.53
**SS2**	cellular FLICE-like inhibitory protein	C-FLIP	414381	2.24
	tumor necrosis factor receptor superfamily, member 8	TNFRSF8	574055	2.08
	death-associated protein kinase 2	DAPK2	100155578	-1.94
	*Rep*: *Mdm4*, *transformed 3T3 cell double minute 4*, *p53 binding protein isoform 1*	MDM4	100512731	-1.94
	programmed cell death 4 (neoplastic transformation inhibitor)	PDCD4	100157112	-1.76
	tumor necrosis factor receptor superfamily, member 1B	TNFRSF1B	100037306	1.64
**H1N1-SS2**	caspase 10, apoptosis-related cysteine peptidase	CASP10	100154896	1.55
	granzyme B (granzyme 2, cytotoxic T-lymphocyte-associated serine esterase 1)	GZMB	100233184	4.29
	Fas ligand (TNF superfamily, member 6)	FASLG	396726	3.33
	BCL2-like 11 (apoptosis facilitator)	BCL2L11	396632	2.40
	caspase 4, apoptosis-related cysteine peptidase	CASP4	100522887	2.36
	tumor necrosis factor receptor superfamily, member 10a	TNFRSF10A	100134832	2.11
	cell death-inducing DFFA-like effector c	CIDEC	100127161	1.96
	BCL2-like 14 (apoptosis facilitator)	BCL2L14	100514901	1.81
	caspase 2, apoptosis-related cysteine peptidase	CASP2	100521118	1.81
	adenomatous polyposis coli	APC	100517932	-1.81
	tumor necrosis factor receptor superfamily, member 8	TNFRSF8	574055	1.75
	*Rep*: *baculoviral IAP repeat-containing protein 2*	BIRC2	100622859	1.75
	*Rep*: *HECT*, *UBA and WWE domain containing 1*	HUWE1	100517442	1.65
	*PREDICTED*: *Sus scrofa tumor necrosis factor alpha-induced protein 8-like*	TNFAIP8	100524014	1.64
	tumor necrosis factor receptor superfamily, member 11b	TNFRSF11B	100049688	-1.63
	DNA-damage-inducible transcript 3	DDIT3	100240743	1.62
	tumor necrosis factor receptor superfamily, member 1B	TNFRSF1B	100037306	1.54
	caspase 3, apoptosis-related cysteine peptidase	CASP3	397244	1.51

The DE genes associated with apoptosis were based on GO term and manual annotation. Manual annotations were listed in italics. Many genes with multiple functions were only listed in one category.

### Validation of microarray data by qRT-PCR

qPCR was performed to validate the expression patterns during infections for specific genes on the same samples used in the microarray analysis. Eight genes (NCF4, CD14, IL17D, CD1D, CD163, IL1R2, TLR9, and TLR2) with different expression in each infection group were selected for qPCR analysis (Table 6). All selected genes were regulated with the same pattern as in the microarray assay through qPCR analysis, although a variation in the fold changes was observed between qPCR and microarray. This phenomenon may arise from the different sensitivities and accuracies between the two techniques. The coincident expression pattern indicated the reliability of the microarray analysis.

**Table 6:** Verification of differentially expressed genes via qRT-PCR.

Genes	H1N1 group			SS2 group			H1N1-SS2 group		
	Microarray fold change	qRT-PCR fold change	qRT-PCR p-value	Microarray fold change	qRT-PCR fold change	qRT-PCR p-value	Microarray fold change	qRT-PCR fold change	qRT-PCR p-value
NCF4	NS	-0.21	< 0.0010	1.64	6.73	< 0.0010	3.34	17.56	< 0.0010
CD14	NS	2.70	< 0.0010	5.23	13.65	< 0.0010	4.96	6.37	< 0.0010
IL17D	NS	2.21	< 0.0010	2.07	4.54	0.019	1.50	15.25	0.0050
CD1D	-1.93	-4.29	< 0.0010	-1.84	-2.16	< 0.0010	3.99	38.90	< 0.0010
CD163	-1.93	-3.62	0.777	3.60	6.01	0.0060	4.98	14.53	0.060
IL1R2	NS	1.49	0.221	14.28	24.73	< 0.0010	4.56	4.12	0.018
TLR9	NS	1.44	0.127	1.58	4.l9	0.0050	2.27	2.16	0.022
TLR2	NS	-3.95	0.042	NS	53.34	0.0070	NS	-1.66	0.83

Fold changes of gene expression in infected group compared with the PBS control. mRNA expressions were measured by real-time RT-PCR. The data represent means ± SEM of triplicate reactions for each gene transcript. The expression level of *GAPDH* was assayed for normalization. Statistical analysis was performed by T-test. NS: No significant change.

## Discussion

Clinically, influenza virus and *S*. *suis* co-infections are common. We also investigated the co-infections in pig farms in the Hubei Province of China using serological test, which exhibited a high co-infection rate (S7 Data). The altered host response after swine influenza virus infection predisposes to secondary bacterial infection because of the complicated mechanisms that would significantly increase the mortality rate. Secondary infection by *S*. *suis* is an example of such case. However, the molecular mechanisms underlying this high mortality rate have not yet been established. The augmented host response is considered as a critical factor for the severe pathogenicity of influenza virus infection in humans [20]. Therefore, a genome expression analysis was performed to obtain comprehensive information on the host responses in the lungs of infected pigs through H1N1, SS2, and H1N1-SS2 infections. The clinical and pathological findings demonstrated that H1N1 and SS2 co-infection enhanced the virulence in pigs. In addition, the transcriptional profiles indicated that co-infection resulted in augmented inflammatory and apoptosis responses, which could account for the increased pathogenecity. The increased virulence of co-infection might also be attributed to the enhanced viral replication or SS2 growth. Increased viral load was observed in lungs and nasal swabs in co-infection group, which might play a role in the virulence. However, the SS2 content in lungs was not significantly different between SS2 and H1N1-SS2 groups in the present study. It suggested that the H1N1 infection might not contribute to the enhanced SS2 growth in pig lungs. Yet, in spite of this, H1N1 infection could damage the epithelia lining of the bronchi and lungs, allowing the SS2 a foothold, and therefore strengthened and disordered the host responses, especially the immune responses in a synergetic manner.

### Inflammatory responses were enhanced in response to H1N1-SS2 co-infection

TLRs are families of pattern-recognition receptors that sense the invasion of pathogenic microorganisms and trigger innate immune responses. Our data showed that several genes in the TLR signaling were significantly but differently regulated in each infection group. For instance, CD14 was markedly upregulated in SS2 and H1N1-SS2 infection groups. CD14 has been identified as an LPS receptor [21], which can bind to the LPS-binding protein (LBP) [22]. LBP catalyzes the transfer of LPS to CD14, which then mediates the transfer of LPS to the membrane, where LPS binds with the TLR4-MD-2 complex, leading to the nuclear factor-kappa B (NF-κB) activation [23, 24]. TLR4 is necessary in this LPS signal transduction process. In fact, CD14 is an essential co-receptor of influenza virus recognition, which is necessary for inflammatory cytokine production induced by influenza virus [25]. TLR4 signaling has also been reported as a key pathway of the acute lung injury induced by H5N1 infection and can be blocked by the TLR4 antagonist, thus protecting the mouse from influenza virus [26, 27]. Based on the microarray data, TLR4 and LBP were slightly but markedly upregulated (1.79- and 1.8-fold, respectively) in the H1N1-SS2 co-infection group (Table 4). However, these genes were not significantly changed in the H1N1 or SS2 infection alone group. MYD88 is an important signaling adapter that contains the toll/IL-1R domain, which can mediate the TLR4 signaling response [28], to elicit a pro-inflammatory response [29]. The results showed that MYD88 was significantly upregulated in the H1N1-SS2 group at 2.32-fold compared with that in the other two infection groups. These results demonstrated that TLR4 signaling have some important roles in the co-infection pathogenesis. Several studies have previously determined that *S*. *suis* was mainly recognized by TLR2, which is associated with CD14 and activates the proinflammatory cytokines. However, the present data showed that TLR2 was not significantly changed in each infection group compared with the control, which suggested that TLR2 may be not a key contributor to the increased virulence by co-infection and this result is consistent with that of a previous study [30]. Thus, the role of TLR4 and its downstream signaling in co-infection are worthy of further investigations.

Cytokine is an important part of the host defensive system that helps eliminate pathogens by recruiting inflammatory cells. However, overabundant cytokine production could cause pathology. In the present study, several cytokines were significantly regulated. For instance, CXCL2, CCL20, and IL2 were significantly upregulated in the H1N1 group (Table 3). In the SS2 infection, IL17D was elevated by 2.07-fold (Table 3), which can stimulate production of IL6, IL8, and GM-CSF production in endothelial cells [31]. In the H1N1-SS2 group, CCL3, CCR1, CXCR4, CX3CL1, IL6ST, and IL17D were differently upregulated (Table 4). Besides, MYD88, NFKB1, TIRAP, TLR4, and TLR9 were upregulated from 1.63- to 2.32-fold in this group. These genes are all involved in the TLR signaling pathway, which can induce and then release pro-inflammatory factors. MAPK, a key pathway that regulates the synthesis of numerous cytokines, chemokines, and other inflammatory mediators [32], was also significantly regulated in the three infection groups. Expectedly, co-infection modified the most number of regulated genes related to MAPK signalling (Fig 3B). The MAPK signaling pathway has been found to be greatly induced and may be one of the key pathways that led to the immune response against *S*. *suis* [33]. Thus, it might suggest that the significant activation of the MAPK pathway plays some important roles in the pro-inflammatory responses caused by co-infection. Interestingly, CD163 was significantly upregulated in the SS2 (3.6-fold) and H1N1-SS2 (4.98-fold) infection groups, however, it was downregulated in H1N1 infection group. CD163 is a macrophage receptor for the bacterial binding [34, 35] and can induce the pro-inflammatory cytokines during infection [35, 36]. In addition, CD163 was associated with SS2 infection [37]. The CD163 upregulation demonstrated that CD163 may have some important roles in inflammatory responses, especially in H1N1-SS2 co-infection. The CD163 function in the co-infection pathogenesis needs further studies. Furthermore, genes related to ROS production were also identified to be upregulated. ROS is involved in many physiological processes, including cell proliferation, apoptosis, and immune and pro-inflammatory responses [38, 39]. Excessive ROS production could cause acute lung injury induced by H5N1 [26, 40]. NADPH oxidase mainly causes the ROS production in inflammatory cells [41, 42], and CYBB is the primary component. CYBB is related to lung inflammation caused by influenza virus [43]. NCF1, NCF2, and NCF4 are all NADPH oxidase components. In the present study, CYBB, NCF1, NCF2, and NCF4 were significantly up-regulated in the H1N1-SS2 group. The increased NADPH oxidase expression implied more ROS production and would lead to oxidative stress, which caused more severe inflammatory damage in H1N1-SS2 group.

To maintain the development and metabolism homeostasis, the host must express anti-inflammatory factors to prevent infection-induced excessive inflammation. The PPAR pathway has an important role in anti-inflammatory response because it can reduce several pro-inflammatory cytokines and chemokines [44]. In the current study, several genes involved in this pathway were upregulated in all three infection groups. In addition, other regulated negative regulators, such as IL13RA1, IL1R2, and IL4R, were detected. This result indicated that the host tried to terminate the over-induced inflammatory responses. TGF-β is another interesting gene, which can act as a global regulator of immunity by controlling the initiation and resolution of inflammatory responses [45, 46]. Previous studies have proven that human H1N1 could induce TGF-β elevation, however, H5N1 downregulated the TGF-β secretion in a mouse model [47, 48]. TGF-β can also protect the host from the lethal influenza virus in a mouse model [49]. Therefore, TGF-β can function as a protective agent against virus invasion. Surprisingly, in our data, TGF-β was downregulated in H1N1 and SS2 group, and no significant change was observed in H1N1-SS2 groups. This result may be attributed to the different animal models used here. The role of TGF-β in immunopathology of infection in pigs remains to be further studied. Based from these results and with the lung pathological lesions, the H1N1-SS2 co-infection is proposed to induce stronger inflammatory responses, which caused more severe inflammatory damage in the lungs.

### Stronger immune responses were induced by H1N1-SS2 co-infection

Cellular immune responses are critical for clearing the influenza virus [50]. NK cells, CD4+ and CD8+ T cells, and neutrophils increased upon influenza virus infection [51]. The increase of the immune cells is not only beneficial for clearing the virus, but may also contribute to pulmonary inflammation, which could cause immune damage and enhance the virulence. In the present study, several genes, as immune cell markers, were moderately upregulated, which contained CD14, CD1D, and CD163, in the SS2 and H1N1-SS2 groups. However, CD163 and CD1D were both downregulated, and CD14 was not significantly changed in H1N1 group. It indicated that the following SS2 infection might enhance or maintain the activation of immune response. However, the composition of the infiltrating immune cells was unknown, and investigating the frequency of immune cells in the lungs and BAL fluid from the H1N1 and SS2 co-infected pigs is highly attractive, because of the important contributions of their inflammatory and immune effect. Antigen processing and presentation is critical for the T cell activation and the adaptive immune response. However, T cells can also contribute to pathology when presented and activated in excess. In the present study, H1N1-SS2 co-infection enhanced several genes expression responsible for the antigen processing and presentation (S8 Data). For instance, SLA-DQA1, which belongs to the MHC class II histocompatibility antigen, was upregulated in H1N1-SS2 group. MHC class II molecules have significant functions in many virus infections in pigs. LGMN was upregulated by 3.47-fold. LGMN, a cysteine protease, was reported to be implicated in human regulatory T cell function [52], and may be involved in the processing of proteins for MHC class II antigen presentation. Specially, KLRC1, an inhibitory receptor of Natural killer (NK) cell, was also upregulated in H1N1-SS2 group. NK cells play an important role in the immune response to viral infections, which can activate dendritic cells and also secret T helper type 1 (Th1) cytokines to augment cytotoxic T cell responses [53]. Previous studies showed that KLRC1 was upregulated on NK cells in HIV and HCV infection [54, 55], and KLRC1 expressing NK cells correlated inversely with HCV RNA [56]. It indicated that KLRC1 might play roles in the H1N1-SS2 co-infection.

Complement is a major effector system of innate immunity, and it also influences the host adaptive immune response to micro-organisms or potential autoantigens [57, 58]. However, accumulating data have suggested that excessive activation of complement is involved in pathogenesis of inflammatory disease, inflammation responses, and acute respiratory distress syndrome [59, 60]. Our data indicated that more genes related to complement system were upregulated, which included C1R, C1S, C5AR1, C6, and KNG1 (S9 Data). It was possible that H1N1-SS2 co-infection triggered much stronger complement response to induce more intense immune responses.

### Robust apoptosis induced by H1N1-SS2 co-infection

Influenza virus has been demonstrated to induce apoptosis, and swine streptococcus has also been reported to induce apoptosis in cultured porcine choroid plexus epithelia cells [61]. The modulation of host cell death pathways may eliminate key immune cells or evade the host defence through pathogen infection. Moreover, the activation or prevention of cell death could be a critical factor for an infection outcome [62]. In the present study, apoptosis was observed in the lungs from all infection groups, and increased robust apoptosis was induced by the H1N1-SS2 co-infection. The microarray analysis results also indicated that more genes involved in apoptosis pathway were regulated. In the H1N1-SS2 group, members of the TNFR superfamily (TNFRSF10A, TNFRSF1B, and TNFRSF8), caspase family (CASP2, CASP3, and CASP4), BCL2-like genes (BCL2L11 and BCL2L14), FASLG, and GZME were upregulated (Table 5). TUNEL test also demonstrated that co-infection led to much higher apoptosis rate compared with separate infections (Fig 5). The impacts of apoptosis on the viral replication vary depending on different virus. Infected cells possibly release the infectious viral particles into micro-environment, which then infect the nearby cells and trigger apoptosis. In this case, the infected nearby cells can host additional virus replication. In the present study, abundant cell apoptosis did not only cause serious lung damage, but also contribute to the dissemination of viruses, which in turn induced stronger pro-inflammatory responses with increased viral load. Therefore, co-infection by H1N1 and SS2 could potentiate virulence by causing severe apoptosis.

In this study, we co-infected SS2 on 3 days after H1N1 inoculation, and killed the pigs on day 6 of the experiment for microarray analysis. The choice of the time was depend on the biological characteristic of H1N1 and SS2. H1N1 infection could cause high-morbidity, but low mortality. The H1N1 infection with 10^7.0^ EID50/mL could cause obvious flu symptoms within 3 days. However, SS2 was highly virulent to pigs. SS2 challenge with 10^6.0^ CFU/mL could lead to severe typical symptoms within 24 hours. In the microarray analysis, many genes reported to play important roles in inflammatory response were not fund to be significantly changed, such as TNF-α, IFN-β, and IL-10. It might be due to the time point selected in our study was relatively later, or the sensitivity of microarray hybridization. The clinical co-infections of influenza virus and SS2 are highly complex, which makes the mechanism of the increased virulence much complex. The factors contributing to the complex may include infection doses of pathogens, the secondary infection time, different pathogens with various virulence, and even the environment factors. Based on this, more studies (studies with more infection times, more infection models, and more pathogens models) are needed to be performed to make the pathogenicity of co-infection much clearer.

## Supporting Information

S1 Data Primers used for qRT-PCR validation. (DOCX)

S2 Data Macroscopic lung lesions in each infection group. On day 6, all pigs were humanely euthanized, and lungs were immediately removed. (A) lungs from each group exhibited different extent lesions. Black arrow indicated bleeding and hyperaemia, Blue arrow indicated necrosis, and Red arrow indicated hyperaemia and pulmonary consolidation. (B) Lung pathology scores were evaluated blindly.
(TIF)

S3 Data Microscopic lung lesions in pigs from control group. Lungs were removed on day 6, and were fixed in formalin and embedded in paraffin, sectioned at 5μm, and stained with hematoxylin and eosin for further histopathologic evaluation.
(TIF)

S4 Data Microscopic lung lesions in pigs from H1N1 group. Lungs were removed on day 6, and were fixed in formalin and embedded in paraffin, sectioned at 5μm, and stained with hematoxylin and eosin for further histopathologic evaluation.
(TIF)

S5 Data Microscopic lung lesions in pigs from SS2 group. Lungs were removed on day 6, and were fixed in formalin and embedded in paraffin, sectioned at 5μm, and stained with hematoxylin and eosin for further histopathologic evaluation.
(TIF)

S6 Data Microscopic lung lesions in pigs from H1N1-SS2 group. Lungs were removed on day 6, and were fixed in formalin and embedded in paraffin, sectioned at 5μm, and stained with hematoxylin and eosin for further histopathologic evaluation.
(TIF)

S7 Data Serological survey of H1N1 and SS2 infection. A total of 376 serum samples from 4 different pig farms were tested for the H1N1 and SS2 antibody by HI and ELISA test respectively.
(DOCX)

S8 Data The DE genes with antigen processing and presentation in each group. The DE genes associated with antigen processing and presentation were assigned based on GO term and manual annotation. Manual annotations were listed in italics. Many genes with multiple functions were only listed in one category.
(DOCX)

S9 Data The DE genes associated with Complement and coagulation cascades in each group. The DE genes associated with Complement and coagulation cascades were assigned based on GO term and manual annotation. Manual annotations were listed in italics. Many genes with multiple functions were only listed in one category.
(DOCX)
