# Supplementary material for: Investigation of Pathogenesis of H1N1 Influenza Virus and Swine Streptococcus suis Serotype 2 Co-Infection in Pigs by Microarray Analysis
Source: PLoS One. 2015 Apr 23;10(4):e0124086. doi: 10.1371/journal.pone.0124086 (PMC4407888; doi:10.1371/journal.pone.0124086)
Supplement: S1 Data — (DOCX) [file pone.0124086.s001.docx]

| **Genes** | **Forward sequences** | **Reverse sequences** |
| --- | --- | --- |
|  |  |  |
| NCF4 | AAGAGCGAGTCCCCACAA | GACGGAGTCCTCATAGAAGC |
| CD14 | GTGGGAGGTGGCAGAGTTCA | CGTCTTCGTCGTCTATTTGG |
| IL17D | CTCCCAGGCAGAACCCAA | CGCCCTTCACTCCACTCA |
| CD1D | GATTCTACCCCAAACCCG | GATTCTACCCCAAACCCG |
| CD163 | CATCATCCTCGGACCCAT | CAGCACAACGACCACCTC |
| IL1R2 | GGTGTTTCTGCCTTCGTC | ACCTTGAGCCTGGTTTGG |
| TLR9 | CCCACGACAGCCGAATAG | GGGGAACAGGGAGCAGAG |
| TLR2 | GACGCTCCCAGATGCCTCCT | GTTGTTGCCTCCAGCTTCCA |
| TLR4 | AACCCAGTGCTGCTTTGAATAG | TGAACAGAAGTGACCCGGAGA |
| MYD88 | CGTCGGATGGTAGTGGTTGTCT | TGGGGAACTCTTTCTTCATTGACT |
| IL-6 | CAAAGCCACCACCCCTAACC | CGTGGACGGCATCAATCTCA |
| IL-8 | TGAGAAGCAACAACAACAGCAG | GTCTGACCAGCACAGGAATGAG |
| CCL2 | TCACCAGCAGCAAGTGTCCT | GCTTCAAGGCTTCGGAGTTT |
| CASP2 | AGGAGGTGTTTCAGCTCTTCGA | GTTCTTTCCATCTTGCTGGTCG |
| CASP3 | AAAGGAGCAGTTTTATTTGCG | AGTTTGGGTTTGCCAGTTAGA |
| BCL2L11 | ATCCCCGCTTTTCATCTTCG | GGTGCTGGGCTCCTGTCTGT |
| FASLG | CCAGCCAAAGGCATACAGAATC | TTAGGCTTGCCTGTTAAGTGGG |
| TNFRSF8 | TGCTGGTAGTTCAGAAGACGGA | GCTTTGACACTTGGAGGTAGGC |
| GAPDH | GGTGAAGGTCGGAGTGAACG | CCATTTGATTTTGGCGGGAT |

**S1 Data Primers for qRT-PCR validation**
