# Supplementary figures and images for: Investigation of Pathogenesis of H1N1 Influenza Virus and Swine Streptococcus suis Serotype 2 Co-Infection in Pigs by Microarray Analysis
Source: PLoS One. 2015 Apr 23;10(4):e0124086. doi: 10.1371/journal.pone.0124086 (PMC4407888; doi:10.1371/journal.pone.0124086)

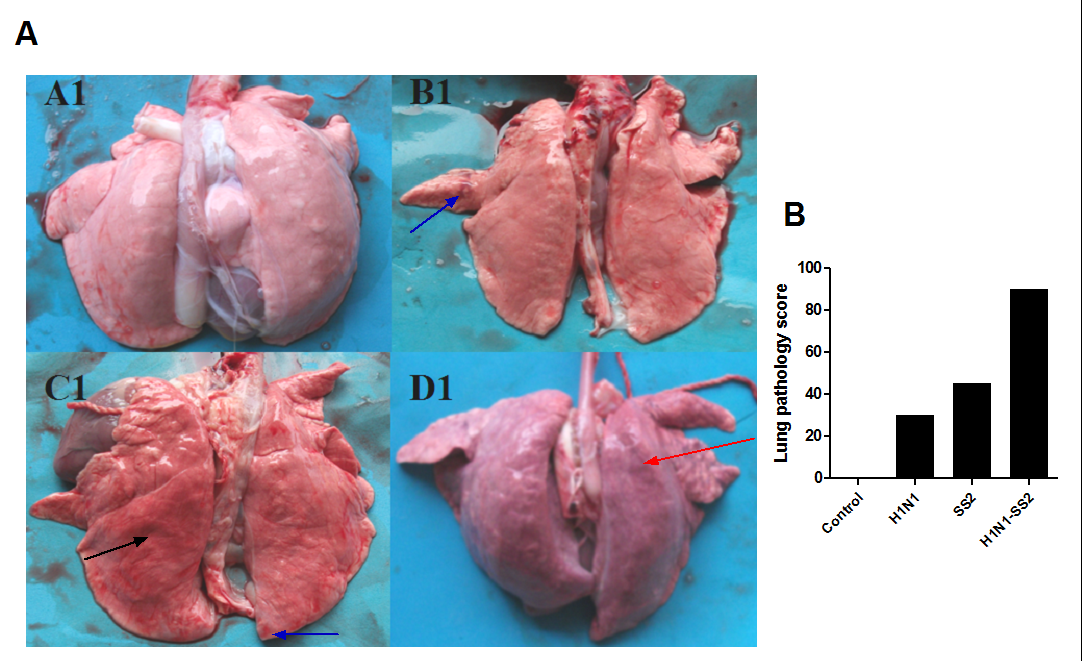

Supplement: S2 Data — On day 6, all pigs were humanely euthanized, and lungs were immediately removed. (A) lungs from each group exhibited different extent lesions. Black arrow indicated bleeding and hyperaemia, Blue arrow indicated necrosis, and Red arrow indicated hyperaemia and pulmonary consolidation. (B) Lung pathology scores were evaluated blindly. (TIF) [file pone.0124086.s002.tif]

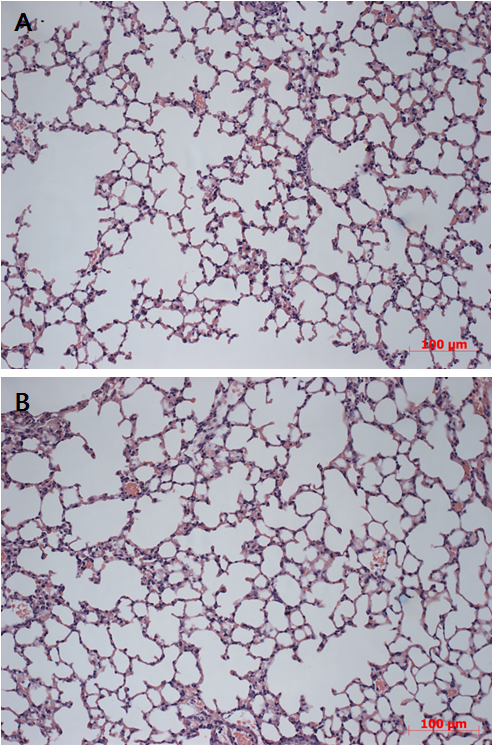

Supplement: S3 Data — Lungs were removed on day 6, and were fixed in formalin and embedded in paraffin, sectioned at 5μm, and stained with hematoxylin and eosin for further histopathologic evaluation. (TIF) [file pone.0124086.s003.tif]

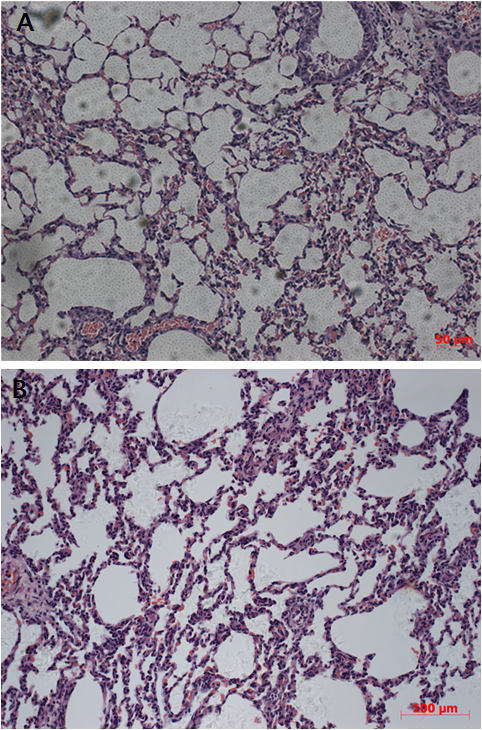

Supplement: S4 Data — Lungs were removed on day 6, and were fixed in formalin and embedded in paraffin, sectioned at 5μm, and stained with hematoxylin and eosin for further histopathologic evaluation. (TIF) [file pone.0124086.s004.tif]

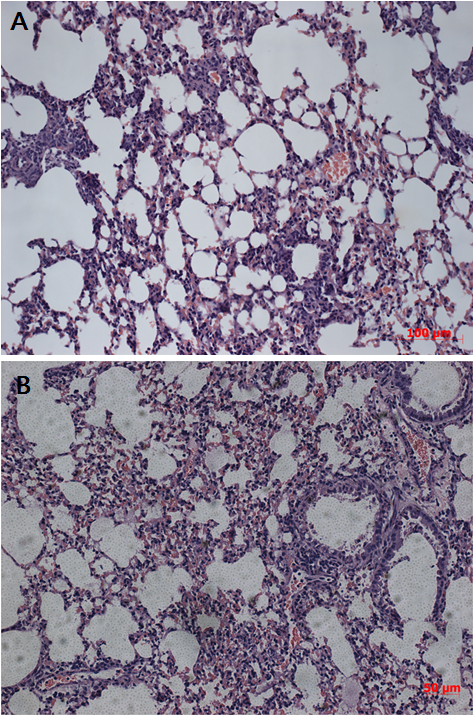

Supplement: S5 Data — Lungs were removed on day 6, and were fixed in formalin and embedded in paraffin, sectioned at 5μm, and stained with hematoxylin and eosin for further histopathologic evaluation. (TIF) [file pone.0124086.s005.tif]

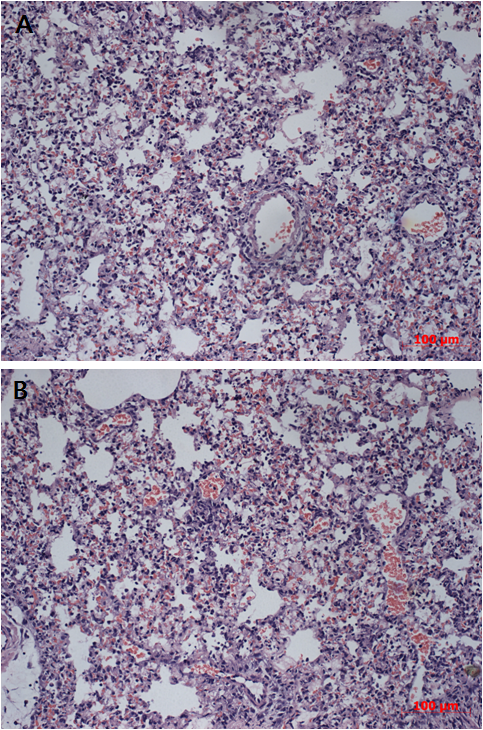

Supplement: S6 Data — Lungs were removed on day 6, and were fixed in formalin and embedded in paraffin, sectioned at 5μm, and stained with hematoxylin and eosin for further histopathologic evaluation. (TIF) [file pone.0124086.s006.tif]
