# Supplementary material for: Investigation of Pathogenesis of H1N1 Influenza Virus and Swine Streptococcus suis Serotype 2 Co-Infection in Pigs by Microarray Analysis
Source: PLoS One. 2015 Apr 23;10(4):e0124086. doi: 10.1371/journal.pone.0124086 (PMC4407888; doi:10.1371/journal.pone.0124086)
Supplement: S7 Data — A total of 376 serum samples from 4 different pig farms were tested for the H1N1 and SS2 antibody by HI and ELISA test respectively. (DOCX) [file pone.0124086.s007.docx]

**S4 Data Serological survey of H1N1 and SS2 infection**

| **Pig farm** | **Antibody positive rate（number, percentage）** | | | | | | |
| --- | --- | --- | --- | --- | --- | --- | --- |
|  | **Total** | **H1N1** | | **SS2** | | **H1N1-SS2** | |
| A | 113 | 46 | 40.71% | 55 | 48.67% | 27 | 23.89% |
| B | 63 | 37 | 58.73% | 35 | 55.56% | 19 | 30.16% |
| C | 69 | 47 | 68.12% | 19 | 27.54% | 15 | 21.74% |
| D | 131 | 87 | 66.41% | 86 | 65.65% | 65 | 49.62% |
| Total | 376 | 217 | 57.71% | 195 | 51.86% | 126 | 33.51% |
