# Supplementary material for: Investigation of Pathogenesis of H1N1 Influenza Virus and Swine Streptococcus suis Serotype 2 Co-Infection in Pigs by Microarray Analysis
Source: PLoS One. 2015 Apr 23;10(4):e0124086. doi: 10.1371/journal.pone.0124086 (PMC4407888; doi:10.1371/journal.pone.0124086)
Supplement: S8 Data — The DE genes associated with antigen processing and presentation were assigned based on GO term and manual annotation. Manual annotations were listed in italics. Many genes with multiple functions were only listed in one category. (DOCX) [file pone.0124086.s008.docx]

**S5 Data The DE genes associated with antigen processing and presentation in each infection group**

| **Group** | **Description** | **Gene symbol** | **Gene ID** | **Fold change** |
| --- | --- | --- | --- | --- |
| **H1N1** | CD1d molecule | CD1D | 100124526 | -1.93 |
|  | CD4 molecule | CD4 | 404704 | 1.58 |
| **SS2** | *PREDICTED: Sus scrofa cathepsin L1-like* | CTSL1 | 396926 | 2.61 |
|  | CD1d molecule | CD1D | 100124526 | -1.84 |
|  | calnexin | CANX | 100519184 | 1.81 |
| **H1N1-SS2** | *PREDICTED: Sus scrofa cathepsin L1-like* | CTSL1 | 396926 | 2.85 |
|  | killer cell lectin-like receptor subfamily C, member 1 | KLRC1 | 100144622 | 2.81 |
|  | *PREDICTED: Sus scrofa similar to legumain* | LGMN | 100154477 | 2.47 |
|  | cathepsin B | CTSB | 100037961 | 1.99 |
|  | protein disulfide isomerase family A, member 3 | PDIA3 | 100156204 | 1.98 |
|  | heat shock 70kDa protein 8 | HSPA8 | 100511890 | 1.97 |
|  | heat shock protein 90kDa alpha (cytosolic), class A member 1 | HSP90AA1 | 397028 | 1.73 |
|  | MHC class II histocompatibility antigen SLA-DQA | HLA-DQA1 | 100153387 | 1.55 |
|  | proteasome (prosome, macropain) activator subunit 2 (PA28 beta) | PSME2 | 397522 | 1.52 |
