# Supplementary material for: Investigation of Pathogenesis of H1N1 Influenza Virus and Swine Streptococcus suis Serotype 2 Co-Infection in Pigs by Microarray Analysis
Source: PLoS One. 2015 Apr 23;10(4):e0124086. doi: 10.1371/journal.pone.0124086 (PMC4407888; doi:10.1371/journal.pone.0124086)
Supplement: S9 Data — The DE genes associated with Complement and coagulation cascades were assigned based on GO term and manual annotation. Manual annotations were listed in italics. Many genes with multiple functions were only listed in one category. (DOCX) [file pone.0124086.s009.docx]

| **Group** | **Description** | **Gene symbol** | **Gene ID** | **Fold change** |
| --- | --- | --- | --- | --- |
| **H1N1** | CD59 molecule, complement regulatory protein | CD59 | 397347 | 2.00 |
| **SS2** | *PREDICTED: Sus scrofa coagulation factor XIII, A1 polypeptide (F13A1), mRNA(111)* | F13A1 | 100153504 | 5.60 |
|  | protein C receptor, endothelial | PROCR | 654289 | 2.86 |
|  | plasminogen activator, tissue | PLAT | 397121 | 2.58 |
|  | CD59 molecule, complement regulatory protein | CD59 | 397347 | 1.54 |
| **H1N1-SS2** | complement component 1, q subcomponent, B chain | C1QB | 100518846 | -3.10 |
|  | complement component 5a receptor 1 | C5AR1 | 100048955 | 2.16 |
|  | kininogen 1 | KNG1 | 396568 | -2.08 |
|  | complement component 6 | C6 | 100037952 | 2.04 |
|  | complement component 1, s subcomponent | C1S | 397274 | 1.71 |
|  | serpin peptidase inhibitor, clade G (C1 inhibitor), member 1 | SERPING1 | 100144304 | 1.70 |
|  | complement component 1, r subcomponent | C1R | 445464 | 1.55 |

**S6 Data The DE genes associated with** **Complement and coagulation cascades**

**In each group**
